# Supplementary material for: Extracting Dynamical Understanding From Neural-Mass Models of Mouse Cortex
Source: Front Comput Neurosci. 2022 Apr 25;16:847336. doi: 10.3389/fncom.2022.847336 (PMC9081874; doi:10.3389/fncom.2022.847336)
Supplement: Supplementary file 1 [file Data_Sheet_1.PDF]

Supplementary Material: Extracting dynamical understanding from neural-mass models of mouse cortex

1 DATA

Structural connectivity

The structural connectivity matrix used here was taken from the Allen Mouse Brain Connectivity Atlas (Oh et al., 2014). We first reduced the original 213×213 connectivity matrix to a 37×37 matrix including our set of cortical areas. We then transformed it into an unweighted, directed connectivity matrix by retained only edges with a p-value less than 0.05 from the regression model fitted by Oh et al. (2014).

Cell-density data

We used excitatory and inhibitory cell-density estimates from Erö et al. (2018). To generate these data, the authors algorithmically generated cell positions and cell types for the entire mouse brain using transcriptional markers from the Allen Mouse Brain Atlas (Lein et al., 2007).

Resting-state fMRI data processing

Mouse fMRI data were preprocessed using an established pipeline for removal of artifacts from the time series (Zerbi et al., 2015; Sethi et al., 2017). Briefly, each 4-dimensional dataset was normalized in a study-specific EPI template (Advanced Normalization Tools [ANTs] v2.1, `picsl.upenn.edu/ANTS`) and fed into MELODIC (Multivariate Exploratory Linear Optimized Decomposition into Independent Components) to perform within-subject spatial independent component analysis (ICA). Thereafter, we applied a mouse-specific classifier (FSL–FIX) to detect and to regress the variance of the nuisance components (Zerbi et al., 2015; Griffanti et al., 2014). This preprocessing pipeline included motion correction, in-plane smoothing with a 0.3 mm kernel, despiking and band-pass filtering (0.01–0.25 Hz). The datasets were then normalized into a mouse MRI template from the Allen Institute, rescaled at 0.2 mm isotropic resolution to match the EPI data size.

2 PERMUTATION TESTING

We aimed to perform a simple statistical test to assess the improvement in FC–FC score resulting from incorporating spatial heterogeneity, via excitatory and inhibitory cell densities, into a coupled network model of W–C neural masses. In particular, our method involves testing for an improved ρ_{FCFC} multiple times, across a range of σ, and therefore has a greater potential to find an improved FCFC, even in the absence of a robust underlying signal. We assessed the statistical significance of the measured result, $\rho_{\mathrm{FCFC}} = 0.60$ (at $\sigma = 0.2$), relative to a null model in which excitatory and inhibitory cell densities were assigned to regions at random. This was done by randomly permuting the rows of the 38×2 (region $\times$ cell density) matrix, and thus does not destroy excitatory—inhibitory correlation structure. We estimated a p-value for the result $\rho_{\mathrm{FCFC}} = 0.60$ as a permutation test relative to a null distribution from 100 randomized simulations, returning the maximum FC–FC score across the range $0 \leq \sigma \leq 1$ in each case. This procedure yielded the estimate $p \approx 0.15$.

3 TABLES

Functional Group	Number	Acronym	Region Name
Somatomotor (Pink)	0	SSs	Supplemental somatosensory area
	1	MOp	Primary motor area
	2	SSp-n	Primary somatosensory area, nose
	3	SSp-ll	Primary somatosensory area, lower limb
	4	SSp-bfd	Primary somatosensory area, barrel field
	5	SSp-m	Primary somatosensory area, mouth
	6	SSp-tr	Primary somatosensory area, trunk
	7	SSp-ul	Primary somatosensory area, upper limb
Medial (Light Blue)	8	PTLp	Posterior parietal association areas
	9	VISam	Anteromedial visual area
	10	VISpm	posteromedial visual area
	11	RSPd	Retrosplenial area, dorsal part
	12	RSPv	Retrosplenial area, ventral part
	13	RSPagl	Retrosplenial area, lateral agranular part
Temporal (Gold)	14	AUDd	Dorsal auditory area
	15	AUDp	Primary auditory area
	16	AUDv	Ventral auditory area
	17	PERI	Perirhinal area
	18	TEa	Temporal association areas
	19	ECT	Ectorhinal area
Visual (Plum)	20	VISal	Anterolateral visual area
	21	VISp	Primary visual area
	22	VISl	Lateral visual area
	23	VISpl	Posterolateral visual area
Anterolateral (Dark Orange)	24	VISC	Visceral area
	25	GU	Gustatory areas
	26	AId	Agranular insular area, dorsal part
	27	AIv	Agranular insular area, ventral part
	28	AIp	Agranular insular area, posterior part
Prefrontal (Green)	29	MOs	Secondary motor area
	30	ACAd	Anterior cingulate area, dorsal part
	31	ORBl	Orbital area, lateral part
	32	PL	Prelimbic area
	33	ORBvl	Orbital area, ventrolateral part
	34	ORBm	Orbital area, medial part
	35	ACAv	Anterior cingulate area, ventral part
	36	ILA	Infralimbic area

Table S1. The 37 cortical regions modeled here. Regions are listed by their ordering used in many plots in the main text, and grouped into six anatomical divisions from Harris et al. (2019), with colors used for annotation in main text figures.

	Regime		
Parameter (units)	Fixed Point	Hysteresis	Limit Cycle
w_{ee} (V s)	12	16	11
w_{ei} (V s)	15	12	10
w_{ie} (V s)	10	10	10
w_{ii} (V s)	8	3	1
b_i (mV)	4	3.7	2.8
τ_e (ms)	10	10	10
τ_i (ms)	10	10	65
a_e (V^{-1})	1	1.3	1
a_i (V^{-1})	1	2	1

Table S2. Parameter values corresponding to the three key model regimes studied in this work. The 'Fixed Point' regime uses parameters modified from Sanz-Leon et al. (2015) (modified to obtain a fixed-point), 'Hysteresis' regime uses parameters from Borisyuk and Kirillov (1992), and the 'Limit Cycle' regime uses parameters from Heitmann et al. (2018).

4 FIGURES

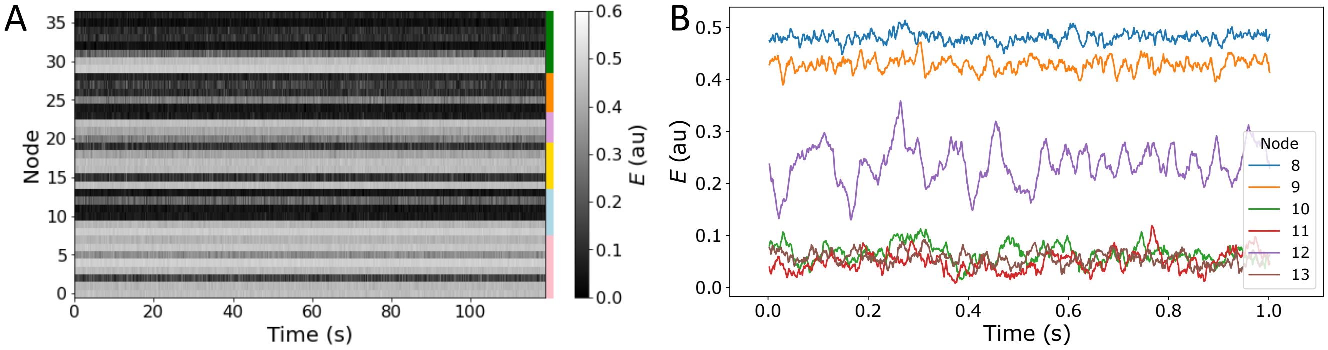

Figure S1. Fixed-point simulation. For the Fixed-Point regime model with the best FC–FC fit ($G = 0.65$, $B_e = 3.3$, FC–FC$= 0.52 \pm 0.03$), we plot: **A** a heat map (carpet plot) of the full time-series simulation, and **B** the final 1 s of simulated dynamics for the six Medial brain regions (numbered 8–13).

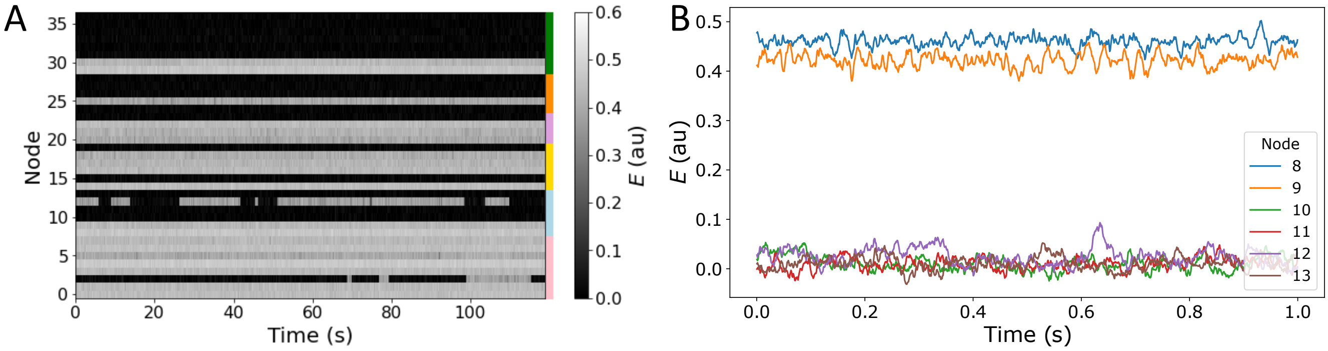

Figure S2. Hysteresis simulation. For the Hysteresis regime model with the best FC–FC fit ($G = 0.35$, $B_e = 3.7$, FC–FC$= 0.50 \pm 0.14$), we plot: **A** a heat map (carpet plot) of the full time-series simulation, and **B** the final 1 s of simulated dynamics for the six Medial brain regions (numbered 8–13). The carpet plot reveals evidence of long-timescale state switching for SSp-n (region 2) and RSPv (region 12).

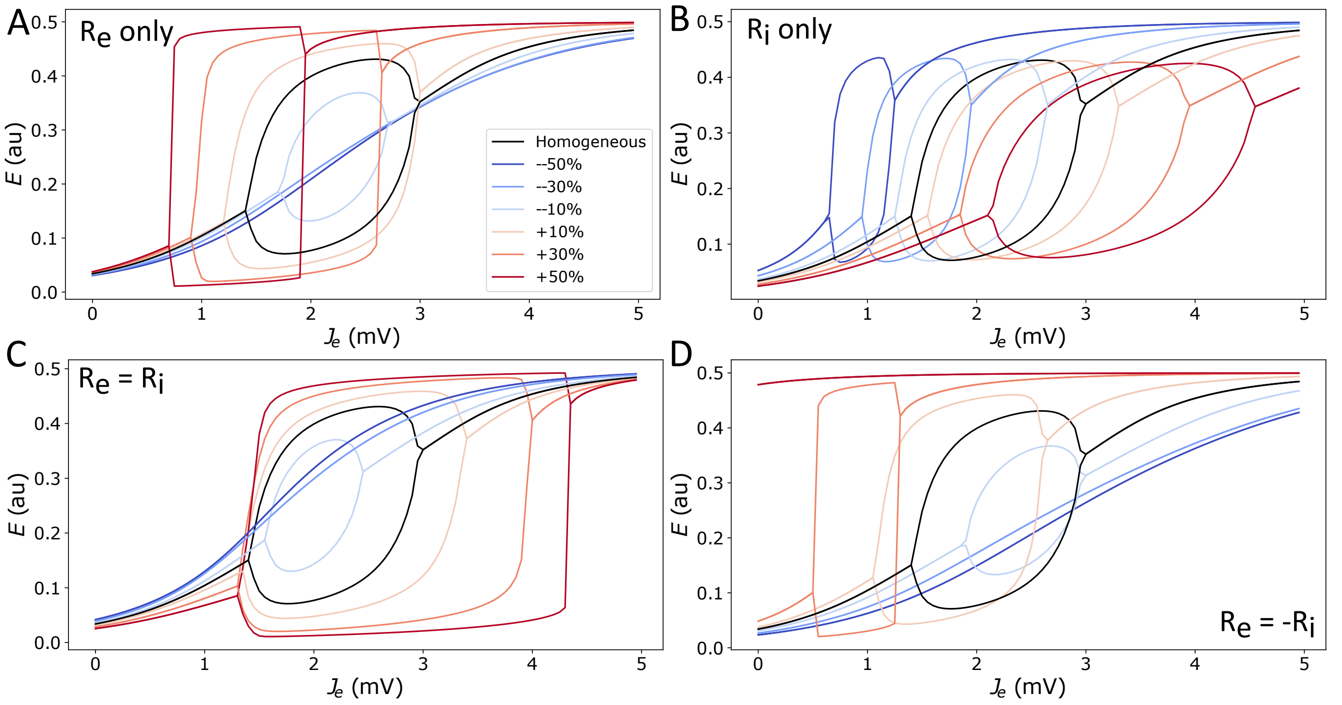

Figure S3. The model's bifurcation structure in the Limit Cycle regime varies substantially when considering perturbations in local excitatory and inhibitory cell density, R_e and R_i, of up to $\pm 50\%$. Instead of $\pm 10\%$ as in Fig. 5, here we plot perturbations of up to $\pm 50\%$. These perturbations can have major effects on the bifurcation structure, including eliminating limit-cycle dynamics altogether.

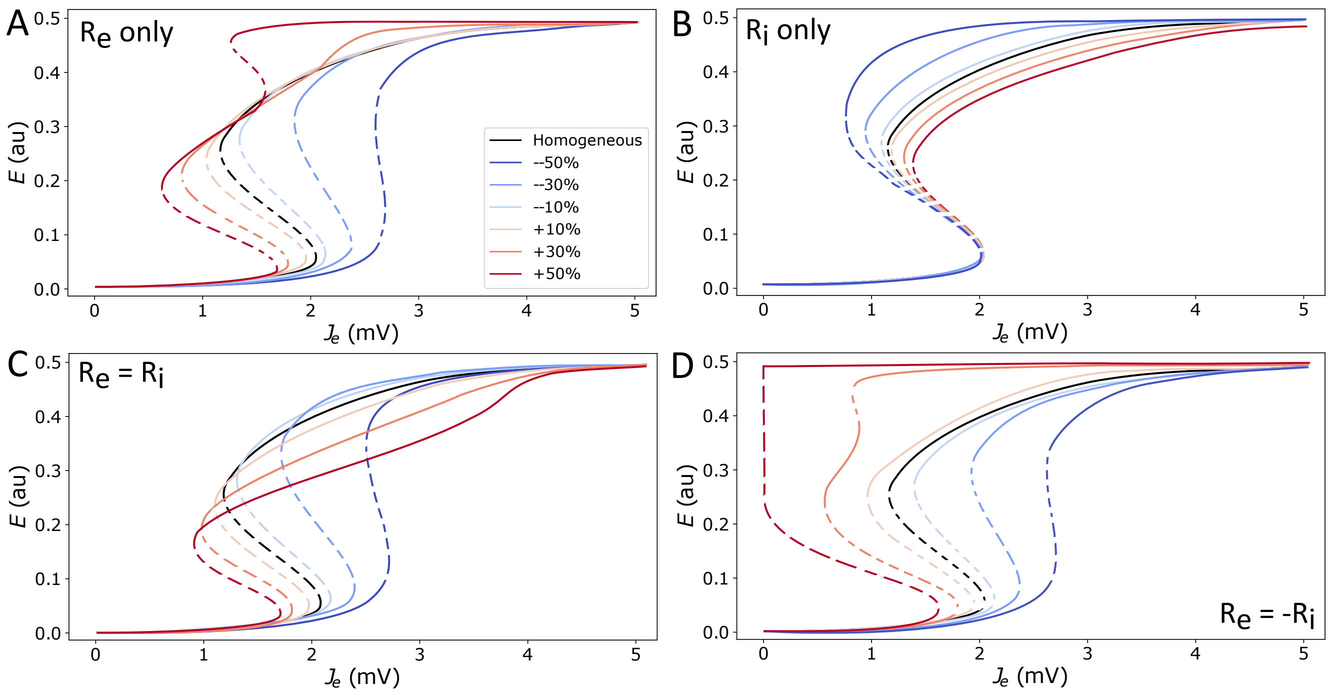

Figure S4. The model's hysteresis bifurcation structure varies substantially when considering perturbations in local excitatory and inhibitory cell density, R_e and R_i, of up to $\pm 50\%$. Of particular interest is the additional multi-stability via a new pair of saddle-node bifurcations (e.g., for $R_e = 0.5$).

REFERENCES

Borisyuk, R. M. and Kirillov, A. B. (1992). Bifurcation analysis of a neural network model. *Biol. Cybern.* 66, 319–325. doi:10.1007/BF00203668

Erö, C., Gewaltig, M.-O., Keller, D., and Markram, H. (2018). A cell atlas for the mouse brain. *Frontiers in Neuroinformatics* 12, e17727. doi:10.3389/fninf.2018.00084

Griffanti, L., Salimi-Khorshidi, G., Beckmann, C., Auerbach, E. J., Douaud, G., Sexton, C. E., et al. (2014). ICA-based artefact removal and accelerated fMRI acquisition for improved resting state network imaging. *NeuroImage* 95, 232–247. doi:10.1016/j.neuroimage.2014.03.034

Harris, J. A., Mihalas, S., Hirokawa, K. E., Whitesell, J. D., Choi, H., Bernard, A., et al. (2019). Hierarchical organization of cortical and thalamic connectivity. *Nature* 575, 195–202. doi:10.1038/s41586-019-1716-z

Heitmann, S., Aburn, M. J., and Breakspear, M. (2018). The Brain Dynamics Toolbox for Matlab. *Neurocomputing* 315, 82–88. doi:10.1016/j.neucom.2018.06.026

Lein, E., Hawrylycz, M. J., Ao, N., Ayres, M., Bensinger, A., Bernard, A., et al. (2007). Genome-wide atlas of gene expression in the adult mouse brain. *Nature* 445, 168–176. doi:10.1038/nature05453

Oh, S. W., Harris, J. A., Ng, L., Winslow, B., Cain, N., Mihalas, S., et al. (2014). A mesoscale connectome of the mouse brain. *Nature* 508, 207–214. doi:10.1038/nature13186

Sanz-Leon, P., Knock, S. A., Spiegler, A., and Jirsa, V. K. (2015). Mathematical framework for large-scale brain network modeling in The Virtual Brain. *NeuroImage* 111, 385–430. doi:10.1016/j.neuroimage.2015.01.002

Sethi, S. S., Zerbi, V., Wenderoth, N., Fornito, A., and Fulcher, B. D. (2017). Structural connectome topology relates to regional BOLD signal dynamics in the mouse brain. *Chaos: An Interdisciplinary Journal of Nonlinear Science* 27, 047405. doi:10.1063/1.4979281

Zerbi, V., Grandjean, J., Rudin, M., and Wenderoth, N. (2015). Mapping the mouse brain with rs-fMRI: An optimized pipeline for functional network identification. *NeuroImage* 123, 11–21. doi:10.1016/j.neuroimage.2015.07.090
